# Liver Injury in Patients Hospitalized for COVID-19: Possible Role of Therapy

**DOI:** 10.3390/vaccines10020192

**Published:** 2022-01-26

**Authors:** Maurizio Gabrielli, Laura Franza, Alessandra Esperide, Irene Gasparrini, Antonio Gasbarrini, Francesco Franceschi, on behalf of GEMELLI AGAINST COVID 2019

**Affiliations:** 1 Department of Emergency, Fondazione Policlinico Universitario A. Gemelli IRCCS, Università Cattolica del Sacro Cuore, 00168 Rome, Italy; cliodnaghfranza@gmail.com (L.F.); alessandraesperide@live.com (A.E.); irene.gasparrini@gmail.com (I.G.); francesco.franceschi@unicatt.it (F.F.); 2 Department of Medical and Surgical Science, Fondazione Policlinico Universitario Agostino Gemelli-IRCCS, Università Cattolica del Sacro Cuore di Roma, Largo A. Gemelli 8, 00168 Rome, Italy; antonio.asbarrini@unicatt.it

**Keywords:** SARS-CoV2, COVID-19, liver, injury, drugs, NIV

## Abstract

Patients with COVID-19 show a high prevalence of liver injury. The pattern of this liver damage is still not fully understood. Different etiopathogenetic factors may concur; from a direct cytopathic effect, once the virus binds to the ACE-2 receptors, to the immune-mediated collateral damage, due to cytokine storm. The presence of pre-existing chronic liver disease is a contributing factor for acute organ damage during SARS-CoV2 infection. Last but not least, treatments probably play a role, also, in determining hepatotoxicity: many of the drugs we have used or are still using to treat COVID-19, combined with non-invasive ventilation, are known to sometimes determine acute liver injury. Although liver damage associated with COVID-19 is often transient and can resolve without any special treatment, it is important to understand the underlying mechanisms, particularly to better treat its more severe forms.

## 1. Introduction

COVID-19 is caused by SARS-CoV-2, a virus belonging to the same family as SARS-CoV-1 and MERS-CoV [1,2,3]. These agents share genomic characteristic and mainly cause respiratory symptoms, even as severe as acute respiratory distress syndrome (ARDS) [2].

SARS-CoV-2 infection is associated with several extra-respiratory symptoms: neurologic, renal, ocular, dermatologic, gastrointestinal, and hepatic [4].

About 3 to 5% of subjects with confirmed SARS-CoV2 infection are admitted to hospital within 14 days due to pneumonia or extra-respiratory complications [5].

In particular, regarding liver involvement, a systematic review and meta-analysis of 128 studies showed that, in COVID-19 patients admitted to hospital, the most common liver abnormalities consisted of elevations of gamma-glutamyl-transferase (GGT, 28%) alanine aminotransferase (ALT, 23%), and aspartate aminotransferase (AST, 23%). In addition, these abnormalities were significantly more common in severe cases when compared with non-severe COVID-19 and are associated with a worse outcome [6].

Liver involvement rises not only in the case of multi-organ failure (MOF), as would be expected, but also in the early stages of the disease [7]. Luckily, most patients recover without the need for specific care [8].

The etiopathogenesis of liver damage in this context is not entirely clear. The increase in cytonecrosis enzymes suggests a direct hepatocellular damage mechanism, possibly through angiotensin converting enzyme 2 (ACE-2), expressed in bile ducts cells and acting as a gateway for the virus [9]. Biopsies showing the presence of viral RNA in liver tissue [10] and signs of hepatocyte apoptosis also strongly support the thesis of direct viral damage [11]. However, ACE-2 expression in normal liver tissue is generally low. A large number of conditions can hijack the expression of ACE-2 receptors, such as hypoxia and fatty liver disease [12,13]. In addition, cytokine storm may play a role in liver damage associated with SARS-CoV2 infection [14].

While the above mechanisms could explain hepatotoxicity through a direct viral damage mechanism, many treatments for COVID-19 also have a high potential for inducing liver injury. This is particularly relevant for patients with comorbidities [15].

Aims of the present article were:

1. to review the potential liver injury associated with pharmacological and non-pharmacological treatments currently in use for hospitalized COVID-19 patients;
2. to assess a reasoned approach to the diagnosis and management of DILI in COVID-19 patients.

## 2. Drugs and Liver Injury

Several drugs can, at least theoretically, cause liver damage (drug-induced liver injury, DILI). DILI is defined by an increase in liver enzymes (hepatocellular necrosis, cholestasis, or both) with and without associated symptoms [16]. According to the updated Roussel Uclaf Causality Assessment Method (RUCAM), the cut-offs for the diagnosis of DILI are as follows: alanine aminotransferase (ALT) > 5 times the upper limit of normal (ULN) and/or alkaline phosphatase (ALP) > 2 times ULN [16,17]. Based on the ratio of serum ALT to ALP values (R-value), DILI pattern may be hepatocellular (≥5), cholestatic (≤2), or mixed (>2 and <5).

Hepatocellular pattern is the most common (>50%) [16,17]. Total bilirubin > 2 times ULN without initial cholestasis is considered a prognostic and not diagnostic criteria since hepatocellular DILI with jaundice is associated to a higher mortality [17].

DILI may occur either through intrinsic or idiosyncratic mechanisms. Intrinsic DILI is predictable and dose-dependent, generally after a brief latency period. The typical drug involved in this kind of DILI is paracetamol (or acetaminophen). Idiosyncratic DILI, the most common form, is unpredictable and its latency period is variable (up to several months) [17,18,19].

DILI ranges from mild to more severe forms, which in 10% of cases require a liver transplant [17,20,21]. A pre-existing chronic liver disease is associated to a worse prognosis, with a mortality three times higher than in those with no pre-existing liver condition [22].

The most commonly involved drugs are acetaminophen and antibiotics [17,23,24]. However, given the complexity of the mechanisms underlying the development of hepatotoxicity, potentially any drug could lead to liver disease [24].

In the present article, we will discuss potential hepatotoxicity of therapies with evidence of efficacy against COVID-19, both pharmacological and not. Table 1 shows the list of all medications approved for COVID-19, the contraindications based on coexisting liver disease, and the risk level of DILI.

### 2.1. Systemic Corticosteroids

Systemic corticosteroids are widely prescribed to outpatients with COVID-19, although there is a lack of data in terms of safety and efficacy in this population. Yet, several trials are available concerning hospitalized patients, the most important being the RECOVERY trial [25]. This large, multicentre study enrolled about 6500 patients admitted to the hospital for COVID-19 infection, randomizing them to receive up to 10 days of dexamethasone (6 mg) or standard of care. Corticosteroid treatment reduced mortality in mechanically ventilated patients and in those requiring oxygen supplementation at enrolment. Systemic corticosteroids other than dexamethasone have been tested in randomized controlled trials as well, but with a much smaller sample size than the one in the RECOVERY trial, and were not sufficient to assess efficacy [26]. When dexamethasone is not available, alternative systemic corticosteroids are methylprednisolone or hydrocortisone, respecting the equivalent total daily dose [27]. Corticosteroids used in combination with immunomodulators such as tocilizumab (see below) showed clinical benefit in some subsets of hospitalized patients with COVID-19 [28].

Prolonged steroid treatment can lead to several adverse events, the most common being hyperglycaemia and secondary infections. DILI is rarely associated with systemic corticosteroid treatment. Conversely, they are commonly administered in the treatment of severe drug-induced liver toxicity [29], even though some authors raise concerns on the safety of corticosteroids in this context [30].

Since hepatotoxicity is uncommon with these drugs, only few case reports are available [31,32,33].

As concerning, specifically, the COVID-19 population, a retrospective study carried out in Hong Kong showed that, in a cohort of 1040 patients, the use of corticosteroids was independently associated with acute liver injury (adjusted OR 3.9, 95% CI 2.1–7.2) [34]. This potential corticosteroids-associated hepatotoxicity could have different possible explanations. First, they are generally administered to patients with severe or critical COVID-19, in which several factors may affect liver function (i.e., several other concomitant drugs with a well-known potential liver toxicity—see below—such as cytokines storm, systemic hypoxia, direct SARS-CoV2 induced liver damage). Second, non-alcoholic fatty liver disease (NAFLD) is the hepatic manifestation of metabolic syndrome, whose essential constituents are among the main risk factors for severe COVID-19 [35]. In this subgroup, corticosteroids may promote non-alcoholic steatohepatitis (NASH), exacerbating insulin resistance, central obesity, diabetes, and hypertriglyceridemia [36].

Unfortunately, RECOVERY and other trials assessing the efficacy of corticosteroid in patients with COVID-19 either did not report drug-related adverse events or showed the most common ones, not mentioning liver damage, thus extensive data in this population is still missing.

### 2.2. Other Immunomodulant Therapies: Anti-IL6, Anti-IL1, Anti-JAK

Patients developing severe forms of COVID-19 generally show strong systemic inflammation associated with increased cytokine release, as suggested by elevated concentration of interleukin (IL)-6, C-reactive protein (CRP), D-dimer, and ferritin [37]. This condition is known as a “cytokine storm” and is able to induce multi-organ damage and long-term consequences [38,39].

Different therapeutic options have been suggested to try to prevent it. Among these, tocilizumab, a recombinant humanized anti-interleukin-6 receptor (IL-6R) monoclonal antibody, is among the most studied [40]. Tocilizumab, combined with dexamethasone, has an indication in hospitalized patients showing a rapid respiratory decompensation due to COVID-19 [28,41]. Indeed, in this subgroup, the use of tocilizumab was associated with a reduction both in mortality and need for intubation [28,41].

The drug has been thoroughly analysed, to evaluate its safety profile. Unsurprisingly, the main reported side effect was immune suppression, yet hepatotoxicity has also been reported [42]. The most common laboratory abnormality is, indeed, elevation of transaminase concentration, which is dose-dependent and generally transient, without significant liver injury or severe complications [42]. Placebo-controlled trials on COVID-19 patients showed a similar safety profile: an increase in liver function tests occurred in 8% with respect to 3% in standard care groups [43]. Evidence of alanine aminotransferase >5 times the upper normal limit is considered a contraindication to the use of tocilizumab, as written in the drug data sheet.

Based on the available evidence, sarilumab, another anti- IL-6R monoclonal antibody, is an alternative to tocilizumab. The safety profile, including liver abnormalities, is comparable to that of tocilizumab [44].

Anakinra, an IL-1 inhibitor, showed significant clinical benefits in patients with COVID-19 and hyperinflammation, and was approved in many countries for the treatment of severe and critical COVID-19, alone or in combination with corticosteroids [45]. Liver damage is not a potential adverse event related to anakinra administration. Increase of liver function tests had a similar incidence in anakinra and placebo groups, in patients either treated for rheumatoid arthritis or receiving the treatment for severe COVID-19 [46,47].

Janus kinase (JAK) inhibitors (baricitinib, tofacitinib, imatinib) showed promising results for reducing mortality and intubation rates and have been approved in many countries for the treatment of the same population of COVID-19 patients as for IL-6 and IL-1 antagonists [48].

Dose-dependent increases in liver enzymes and bilirubin during treatment with JAK-inhibitors have been reported in less than 1% of patients in clinical studies in patients with an indication other than COVID-19. In addition, no severe DILI cases were reported. It should be underlined that many of the patients with evidence of liver function test abnormalities were receiving concomitant hepatotoxic drugs, the most common being methotrexate or isoniazid [49]. Randomized-controlled trials on COVID-19 patients showed a similar incidence of adverse events between groups. In addition, they did not report liver damage among safety outcomes [50].

### 2.3. Antivirals: Remdesivir

Several antiviral agents have been used to treat SARS-CoV-2 infection, with variable degrees of success. The only drug of this class approved and recommended at present is remdesivir. It is an active inhibitor of viral RNA-dependent RNA polymerases. Dose to treat COVID-19 was 200 mg on day 1, then 100 mg daily from a minimum of 4 to a maximum of 9 days. Remdesivir appears to be effective within 10 days from symptom onset in patients admitted to the hospital for COVID-19, who require oxygen supplementation. No benefit was observed in more severe cases, such as patients on high-flow oxygen, non-invasive ventilation (NIV), and mechanical ventilation [51,52].

Adverse effects of remdesivir include serum aminotransferase elevation among others. Liver function tests should be obtained before and during remdesivir administration. The drug should be discontinued if ALT levels increase to >10 times the upper normal limit or there is ALT elevation associated with signs or symptoms of liver inflammation [53].

The elevation of serum aminotransferase without other significant symptoms was reported in up to 9% of patients. It is generally asymptomatic, fully reversible, and not associated with jaundice [18].

Interestingly, as for randomized placebo-controlled trials on the use of remdesivir in COVID-19 patients, serious adverse events were significantly less common in patients treated with the drug when compared with the placebo group. In particular, remdesivir was associated with a lower occurrence of serum aminotransferase elevation (OR 0.76, CI 0.60–0.96) [53]. This suggests that the risk of liver damage is not a real cause of concern for remdesivir use in COVID-19 patients, for whom the drug is indicated.

### 2.4. Low-Molecular-Weight Heparins

Anticoagulants are widely used to treat patients with COVID-19. Low-molecular-weight heparins (LMWH) are by far the most commonly used ones, given the evidence in terms of significant impact on both morbidity and mortality. Literature of the last forty years has shown that the main side effects of these drugs are bleeding, thrombocytopenia, and hepatotoxicity [54]. The frequency of LMWH induced DILI ranges between 4.3–13%, thus representing the most common cause of hepatotoxicity among anticoagulants [55,56]. Risk factors for developing heparin-associated hepatotoxicity and the underlying mechanism are unknown. From a histopathological standpoint, there is an absence of serum plasma cells and eosinophils, suggesting either a direct toxic effect on the hepatocytes or hypersensitivity reactions as the most plausible pathogenic mechanism [57]. Liver enzyme elevation generally occurs between 5 and 8 days after initiation of heparin and normalizes or improves within 2 weeks of drug cessation [58].

No specific data are available about liver injury during heparin treatment in patients with COVID-19. Four large randomized-controlled trials on different dosages of heparin in patients admitted to hospital for COVID-19 are available. None of them included liver injury among safety outcomes [59,60,61,62].

### 2.5. Symptomatic Medications: NSAIDs and Paracetamol

At present, there is no strong evidence in favour or against the use of non-steroidal anti-inflammatory drugs (NSAIDs) in patients with COVID-19 [63]. For this reason, EMA did not give any strong recommendation against the use of these drugs, even though it suggests using paracetamol (acetaminophen) as a first line to treat fever and pain [64].

NSAIDs exert their properties through inhibition of cyclooxygenase (COX)-1 and -2 and the most common side effects of these drugs are primarily gastrointestinal and renal. Yet, literature reports cases of liver toxicity: in a retrospective cohort study, NSAIDs were deemed responsible of over a third of all DILIs reported in a single institution [65].

Paracetamol has both antipyretic and analgesic effects, probably due to inhibition of cyclooxygenases (COX-1, COX-2, and COX-3) and modulation of the endocannabinoid system and serotonergic pathways [66]. Paracetamol is a well-known cause of dose-dependent DILI; its overdose is not a rare event, it can sometimes be intentional, and it is difficult to manage, with potentially fatal consequences [23]. In adults, 12 g is the average dose for the onset of severe hepatotoxicity: peak toxicity usually occurs in about 48–96 h [67]. The underlying toxicity mechanism is a dose-dependent direct damage, through acetaminophen metabolite NAPQI. Studies also suggest that, even at lower doses than the ones necessary for an overdose, paracetamol carries a risk of hepatoxicity that is not inferior to that associated to therapeutic doses of NSAIDs [68]. It is unknown if it depends on metabolic differences, accumulation of paracetamol metabolites or immune-mediated mechanisms.

To date no specific data are available concerning hepatotoxicity or other adverse events associated with a symptomatic use of NSAIDs and paracetamol in patients with COVID-19.

### 2.6. Antibiotics

While using antivirals has a clear rationale in the context of COVID-19 infection, the rationale behind the use of antibiotics is not so obvious. About 75% of patients with COVID-19 receive antibiotics, which is surprising when compared to the estimated bacterial co-infection (8.6%). This phenomenon appears to affect both outpatient and hospitalized patients [69]. Several antibiotics are known to be able to induce hepatotoxicity, the most common being amoxicillin/clavulanate [70]. Other antibiotics potentially causing DILI are trimethoprim/sulphamethoxazole, tetracycline, and clindamycin [71].

Azithromycin deserves a separate discussion, since, particularly at the beginning of the pandemic, it was widely used. This antibiotic was met enthusiastically more for its antiviral and anti-inflammatory properties, rather than its microbicide effect. Azithromycin has, indeed, been demonstrated to reduce inflammatory cytokines (particularly IL-6, IL-8, TNF-α), modulate T-helper functions and reduce oxidative stress [71,72]. A French study suggested that azithromycin was useful in combination with hydroxychloroquine in reducing the burden of COVID-19 disease [73,74], but it was soon disputed [75]. To date no national or international drug regulating agency recommends using azithromycin during COVID-19 disease [76,77].

One of the main concerns in using azithromycin is its cardiac toxicity, particularly when used in association with hydroxychloroquine [78], yet it is interesting to underline that azithromycin is also associated to hepatotoxicity [79,80]. Different studies have concentrated on the characteristics of azithromycin-related hepatotoxicity and showed that patients who already suffered from liver disease were more at risk of developing serious complications [22].

## 3. Non-Pharmacological Treatments: Non-Invasive Ventilation

For the aim of this review, we will use the term non-invasive ventilation (NIV) to indicate both pressure support ventilation and continuous positive airway pressure. All around the world, also outside the intensive care unit, NIV has been largely employed to treat patients with acute respiratory failure due to SARS-CoV2 infection. It was effective in 74% of patients, whereas 26% were non-responders. This data is even of greater importance when remembering that about 23% of COVID-19 patients treated by NIV received ‘do-not-intubate’ orders [81]. In addition, NIV seems more effective than high-flow nasal cannula oxygenation, especially in patients with COVID-19 and moderate-to-severe respiratory failure (PaO_2_/FiO_2_ ≤ 200) [81,82].

Positive end-expiratory pressure (PEEP) is crucial in treating COVID-19 related acute respiratory failure. PEEP values between 5 and 15 cmH2O lead to oxygenation improvement not only increasing alveolar recruitment but also acting on abnormalities of the lung circulation [83]. At the beginning of the pandemic, the median level of PEEP was higher (mean 14, range 12–16 cmH2O) [84]. Later, particularly during the second wave, PEEP levels substantially decreased, because of a more accurate knowledge of the peculiar physiopathology of ARDS associated with COVID-19 [85].

Higher PEEP levels may determine liver damage through different mechanisms reduced hepatic venous outflow thus leading to hepatic congestion [86], and liver inflammation (as expressed by an increased number of neutrophils and lymphocytes in the liver sinusoids), in particular [87]. In addition, during septic shock, higher PEEP may affect cardiac output, decreasing the hepatic arterial flow, thus enhancing arterial dysfunction [88]. Higher PEEP values were associated to hepatic dysfunction both in animal models and in critically ill patients [87,89].

At present, no specific data are available about the impact of NIV on liver function, in patients with COVID-19. However, it is plausible that in severe or critical COVID-19, the systemic hyperinflammation per se affects liver function and acts as fuel for acute liver injury by multiple triggers as drugs and NIV [13].

## 4. Diagnosis and Management of DILI in COVID-19 Patients

The diagnosis of DILI is always challenging, even more so in patients admitted to hospital for COVID-19. However, even in this case, DILI remains a diagnosis by elimination [17,18,19,24].

A thorough patient history should be collected, including history of chronic liver disease or other comorbidities, alcohol abuse, either chronic or acute, and the full list of medications and their dosage assumed in the last 6 months [17,18,19,24]. The consultation of the web site LiverTox (https://www.ncbi.nlm.nih.gov/books/NBK547852/, accessed on 23 November 2021) is of great importance, since it describes the most common patterns of DILI for hundreds of different drugs [80]. We should always also ask also for herbal and dietary supplement use, which are typically self-prescribed and gained further popularity at the time of COVID-19. Many patients search the web in hopes of finding “something natural”, to protect themselves from the disease, and buy directly online several products, often of uncertain origin and not all always safe [90,91].

**Figure 1:**
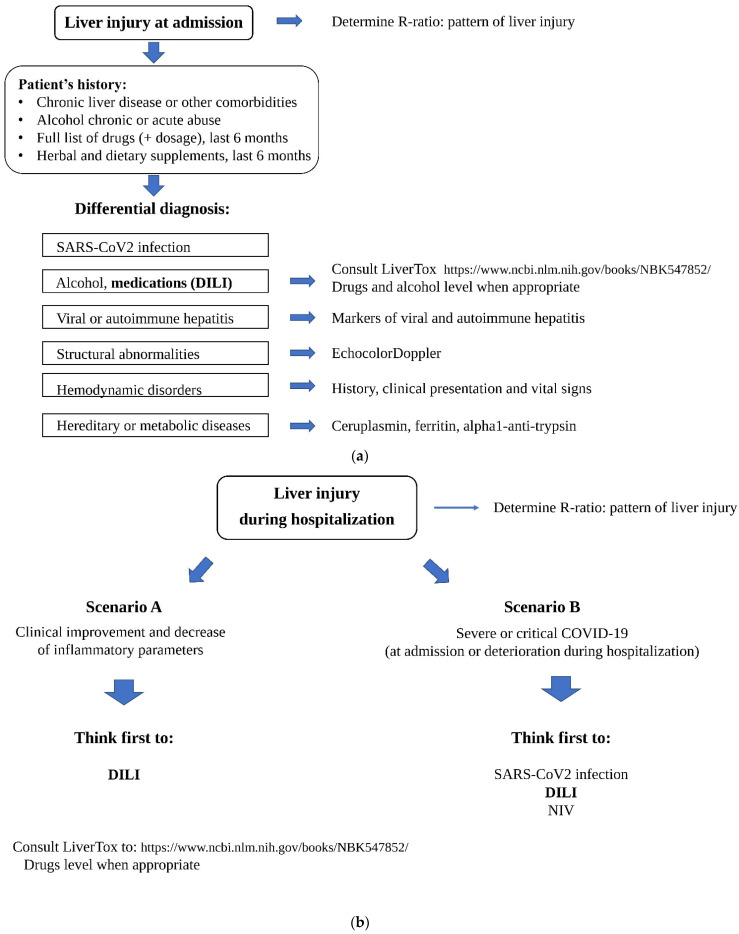
(**a**) Suggestion of a flow chart to assess DILI in patients with COVID-19 at admission. (**b**) Suggestion of a flow chart to assess DILI in patients with COVID-19 during hospitalization.

In addition, we should always make a differential diagnosis with other potential causes of liver damage, such as viral or autoimmune hepatitis, structural or hemodynamic disorders, and hereditary diseases (i.e., Wilson disease and alpha-1 antitrypsin deficiency) (see Figure 1a for details) [17,18,19,24].

However, in the specific case of COVID-19, we should first consider more plausible aetiologies, particularly acute liver damage associated to SARS-CoV2 infection [8,14]. Based on available literature data, it is probably due to the immune-inflammatory response against the virus, sometimes leading to a real cytokine storm, more than to direct entrance of the microorganism inside the liver [8,14].

Yet, when liver test abnormalities occur later during the hospitalization, we should consider two principal possibilities (see Figure 1b for details). If the patient has improved clinically and inflammatory parameters are decreasing, the most plausible aetiology is DILI, associated to at least one of the medications administered to treat COVID-19. In the case of presentation as (or clinical deterioration to) severe acute respiratory failure, it could be extremely complicated to identify a specific aetiology, because multiple possible mechanisms may play a role in determining the damage, from acute liver injury associated with COVID-19 to NIV, and DILI.

In general, liver biopsy is considered not necessary to establish a diagnosis of DILI [17,18,19,24]. This is even more true during COVID-19 infection, since liver biopsy may be associated with a significant higher risk of complications, due to treatment in some cases, particularly when the patient is being treated with intermediate-dose or therapeutic-dose heparin, and/or presents with COVID-19-associated coagulopathy [59,60,61,62,92].

Acute liver injury during hospitalization for COVID-19 is often mild and transient, and acute liver failure is a rare event. Indeed, in most cases it does not require specific therapy to recover [17,18,19,24].

There are no specific guidelines for the management of DILI in COVID-19. In general, the most effective treatment of suspected DILI is discontinuation of the medication, which leads to spontaneous recovery in about 90% of cases. The only available antidote remains N-acetylcysteine for paracetamol overdose. Treatment of acute liver failure follows the general well known flow charts for DILI, including support therapies and considering urgent transplantation [17,18,19,24].

Given to the importance of therapy with systemic corticosteroids and other immune-modulators, heparin, remdesivir, NIV, when indicated for ARDS or other complications, the decision to discontinue one or more of them should be thoroughly examined. The problem hardly arises in the case of drugs such as tocilizumab or sarilumab (generally one or two infusions) or remdesivir (generally 5 days). However, when the suspicion of DILI involves other treatments t need prolonged administration, their discontinuation is mandatory if acute liver failure occurs, and it should be evaluated if ALT levels increase to >10 times the upper normal limit or there is ALT elevation associated with clinical worsening attributable to liver inflammation.

Since COVID-19 patients, especially those hospitalized, are particularly prone to liver injury, symptomatic treatment with NSAIDs and/or paracetamol should be administered with caution. In addition, widespread administration of an “antibiotic coverage” should be avoided and considered only if there is strong suspicious of bacterial superinfection, also according to antibiotic stewardship rules [93]. In this case, if possible, we should choose antibiotic schemes recommended by guidelines for hospital-acquired pneumonia, possibly preferring those with lower incidence of liver injury [94].

## 5. Conclusions

While we still do not completely understand the ways through which liver damage takes place during COVID-19 infection, it is important to remember that liver damage can significantly affect the outcome of patients. There are different possible actions we can take to try to reduce liver damage during the infection. First, we need to screen patients for signs of liver dysfunction before starting therapy, to adjust if necessary. We also should avoid using not recommended therapies, given the potential liver damage they could induce. Lastly, we should continue to monitor liver functionality throughout the disease and the course of therapy, to be ready to take action if necessary.

## Figures and Tables

**Table 1:** List of all drugs approved for COVID-19, their liver contraindications, risk level of DILI and its most common pattern.

Drug	Liver Contraindications	Risk Level of DILI	DILI Pattern
systemic corticosteroids	Generic caution in liver failure	+	hepatocellular or mixed
tocilizumab	ALT > 5 times URL	++	hepatocellular
sarilumab	ALT > 1.5 times URL	++	hepatocellular
anakinra	Not recommended in severe liver failure (Child-Pugh C)	+	hepatocellular
baricitinib, tofacitinib, imatinib	Not recommended in severe liver failure (Child-Pugh C)	+	hepatocellular
remdesivir	ALT > 5 times URL	++	hepatocellular
low-molecular-weight heparins	Generic caution in liver failure	+	hepatocellular
NSAIDs	Contraindicated in severe liver failure (Child-Pugh C), caution in mild or moderate liver failure (indication to use low dosage)	++	hepatocellular
paracetamol	Contraindicated in severe liver failure (Child-Pugh C), caution in mild or moderate liver failure (indication to use low dosage)	++++	hepatocellular
antibiotics amoxicillin-clavulanate trimethoprim/sulfamethoxazole azitromicin		+++ ++ ++	cholestatic cholestatic mixed

## References

[1] Pollard C.A., Morran M.P., Nestor-Kalinoski A.L. (2020). The COVID-19 pandemic: A global health crisis. Physiol. Genom..

[2] Li X., Ma X. (2020). Acute respiratory failure in COVID-19: Is it “typical” ARDS?. Crit. Care.

[3] Rastogi M., Pandey N., Shukla A., Singh S.K. (2020). SARS coronavirus 2: From genome to infectome. Respir. Res..

[4] Lai C.-C., Ko W.-C., Lee P.-I., Jean S.-S., Hsueh P.-R. (2020). Extra-respiratory manifestations of COVID-19. Int. J. Antimicrob. Agents.

[5] Ahmed J., Rizwan T., Malik F., Akhter R., Malik M., Ahmad J., Khan A.W., Chaudhary M.A., Usman M.S. (2020). COVID-19 and Liver Injury: A Systematic Review and Meta-Analysis. Cureus.

[6] Kumar-M P., Mishra S., Jha D.K., Shukla J., Choudhury A., Mohindra R., Mandavdhare H.S., Dutta U., Sharma V. (2020). Coronavirus disease (COVID-19) and the liver: A comprehensive systematic review and meta-analysis. Hepatol. Int..

[7] Fu T., Deng M., Wang X., Yang F. (2020). Predictor of poor prognosis of COVID-19 patients–liver injury. Expert Rev. Gastroenterol. Hepatol..

[8] Zhang C., Shi L., Wang F.-S. (2020). Liver injury in COVID-19: Management and challenges. Lancet Gastroenterol. Hepatol..

[9] Chai X., Hu L., Zhang Y., Han W., Lu Z., Ke A., Zhou J., Shi G., Fang N., Fan J. (2020). Specific ACE2 Expression in Cholangiocytes May Cause Liver Damage After 2019-nCoV Infection. bioRxiv.

[10] Qi F., Qian S., Zhang S., Zhang Z. (2020). Single cell RNA sequencing of 13 human tissues identify cell types and receptors of human coronaviruses. Biochem. Biophys. Res. Commun..

[11] Wang Y., Liu S., Liu H., Li W., Lin F., Jiang L., Li X., Xu P., Zhang L., Zhao L. (2020). SARS-CoV-2 infection of the liver directly contributes to hepatic impairment in patients with COVID-19. J. Hepatol..

[12] Paizis G., Tikellis C., Cooper M.E., Schembri J.M., Lew R.A., Smith I.A., Shaw T., Warner F.J., Zuilli A., Burrell L.M. (2005). Chronic liver injury in rats and humans upregulates the novel enzyme angiotensin converting enzyme 2. Gut.

[13] Testino G., DI Biagio A., Fagoonee S., Pellicano R. (2021). SARS-CoV-2, alcohol consumption and liver injury. Minerva Med..

[14] Effenberger M., Grander C., Grabherr F., Griesmacher A., Ploner T., Hartig F., Bellmann-Weiler R., Joannidis M., Zoller H., Weiss G. (2021). Systemic inflammation as fuel for acute liver injury in COVID-19. Dig. Liver Dis..

[15] Ferron P.J., Gicquel T., Mégarbane B., Clément B., Fromenty B. (2020). Treatments in Covid-19 patients with pre-existing metabolic dysfunction-associated fatty liver disease: A potential threat for drug-induced liver injury?. Biochimie.

[16] Benichou C., Danan G., Flahault A. (1993). Causality assessment of adverse reactions to drugs—II. An original model for validation of drug causality assessment methods: Case reports with positive rechallenge. J. Clin. Epidemiol..

[17] Sandhu N., Navarro V. (2020). Drug-Induced Liver Injury in GI Practice. Hepatol. Commun..

[18] Katarey D., Verma S. (2016). Drug-induced liver injury. Clin. Med..

[19] Davern T.J. (2012). Drug-induced liver disease. Clin. Liver Dis..

[20] Teschke R. (2019). Idiosyncratic DILI: Analysis of 46,266 Cases Assessed for Causality by RUCAM and Published From 2014 to Early 2019. Front. Pharmacol..

[21] Kuna L., Bozic I., Kizivat T., Bojanic K., Mrso M., Kralj E., Smolic R., Wu G.Y., Smolic M. (2018). Models of Drug Induced Liver Injury (DILI)–Current Issues and Future Perspectives. Curr. Drug Metab..

[22] Chalasani N., Bonkovsky H.L., Fontana R., Lee W., Stolz A., Talwalkar J., Reddy K.R., Watkins P.B., Navarro V., Barnhart H. (2015). Features and Outcomes of 899 Patients With Drug-Induced Liver Injury: The DILIN Prospective Study. Gastroenterology.

[23] Larson A.M. (2007). Acetaminophen hepatotoxicity. Clin. Liver Dis..

[24] Andrade R.J., Aithal G.P., Björnsson E.S., Kaplowitz N., Kullak-Ublick G.A., Larrey D., Karlsen T.H., European Association for the Study of the Liver (2019). EASL Clinical Practice Guidelines: Drug-induced liver injury. J. Hepatol..

[25] RECOVERY Collaborative Group (2021). Dexamethasone in Hospitalized Patients with Covid-19. N. Engl. J. Med..

[26] Jeronimo C.M.P., Farias M.E.L., Val F.F.A., Sampaio V.S., Alexandre M.A.A., Melo G.C., Safe I.P., Borba M.G.S., Netto R.L.A., Maciel A.B.S. (2020). Methylprednisolone as Adjunctive Therapy for Patients Hospitalized With Coronavirus Disease 2019 (COVID-19; Metcovid): A Randomized, Double-blind, Phase IIb, Placebo-controlled Trial. Clin. Infect. Dis..

[27] Sterne J.A., Murthy S., Diaz J.V., Slutsky A.S., Villar J., Angus D.C., Annane D., Azevedo L.C., Berwanger O., Cavalcanti A.B. (2020). Association Between Administration of Systemic Corticosteroids and Mortality Among Critically Ill Patients With COVID-19: A Meta-analysis. JAMA.

[28] Chalmers J., Abo-Leyah H., Loftus H., Spears M. (2021). Tocilizumab in patients admitted to hospital with COVID-19 (RECOVERY): A randomised, controlled, open-label, platform trial. Lancet.

[29] Cortes M.G., Robles-Diaz M., Stephens C., Ortega-Alonso A., Lucena M.I., Andrade R.J. (2020). Drug induced liver injury: An update. Arch. Toxicol..

[30] Hu P.-F., Xie W.-F. (2018). Corticosteroid therapy in drug-induced liver injury: Pros and cons. J. Dig. Dis..

[31] Hori T., Onishi Y., Kamei H., Kurata N., Ishigami M., Ishizu Y., Ogura Y. (2016). Fibrosing cholestatic hepatitis C in post-transplant adult recipients of liver transplantation. Ann. Gastroenterol..

[32] Cheung A.Y., Patel S., Kurji K.H., Sarnicola E., Eslani M., Govil A., Holland E.J. (2018). Ocular Surface Stem Cell Transplantation for Treatment of Keratitis–Ichthyosis–Deafness Syndrome. Cornea.

[33] Tanaka Y., Uchida T., Yamaguchi H., Kudo Y., Yonekawa T., Nakazato M. (2019). Fulminant hepatitis and elevated levels of sIL-2R in thyroid storm. Endocrinol. Diabetes Metab. Case Rep..

[34] Yip T.C.-F., Lui G.C.-Y., Wong V.W.-S., Chow V.C.-Y., Ho T.H.-Y., Li T.C.-M., Tse Y.-K., Hui D.S.-C., Chan H.L.-Y., Wong G.L.-H. (2020). Liver injury is independently associated with adverse clinical outcomes in patients with COVID-19. Gut.

[35] Woods C., Hazlehurst J.M., Tomlinson J.W. (2015). Glucocorticoids and non-alcoholic fatty liver disease. J. Steroid Biochem. Mol. Biol..

[36] Farrell G.C. (2002). Drugs and Steatohepatitis. Semin. Liver Dis..

[37] Zhou F., Yu T., Du R., Fan G., Liu Y., Liu Z., Xiang J., Wang Y., Song B., Gu X. (2020). Clinical course and risk factors for mortality of adult inpatients with COVID-19 in Wuhan, China: A retrospective cohort study. Lancet.

[38] Ragab D., Eldin H.S., Taeimah M., Khattab R., Salem R. (2020). The COVID-19 Cytokine Storm; What We Know So Far. Front. Immunol..

[39] Fajgenbaum D.C., June C.H. (2020). Cytokine Storm. N. Engl. J. Med..

[40] Quirch M., Lee J., Rehman S. (2020). Hazards of the Cytokine Storm and Cytokine-Targeted Therapy in Patients with COVID-19: Review. J. Med Internet Res..

[41] Kyriakopoulos C., Ntritsos G., Gogali A., Milionis H., Evangelou E., Kostikas K. (2021). Tocilizumab administration for the treatment of hospitalized patients with COVID-19: A systematic review and meta-analysis. Respirology.

[42] Sheppard M., Laskou F., Stapleton P.P., Hadavi S., Dasgupta B. (2017). Tocilizumab (Actemra). Hum. Vaccines Immunother..

[43] Salvarani C., Dolci G., Massari M., Merlo D.F., Cavuto S., Savoldi L., Bruzzi P., Boni F., Braglia L., Turrà C. (2021). Effect of Tocilizumab vs. Standard Care on Clinical Worsening in Patients Hospitalized With COVID-19 Pneumonia: A Randomized Clinical Trial. JAMA Intern. Med..

[44] Ngamprasertchai T., Kajeekul R., Sivakorn C., Ruenroegnboon N., Luvira V., Siripoon T., Luangasanatip N. (2021). Efficacy and Safety of Immunomodulators in Patients with COVID-19: A Systematic Review and Network Meta-Analysis of Randomized Controlled Trials. Infect. Dis. Ther..

[45] Kyriakoulis K.G., Kollias A., Poulakou G., Kyriakoulis I.G., Trontzas I.P., Charpidou A., Syrigos K. (2021). The Effect of Anakinra in Hospitalized Patients with COVID-19: An Updated Systematic Review and Meta-Analysis. J. Clin. Med..

[46] Fleischmann R.M., Schechtman J., Bennett R., Handel M.L., Burmester G.R., Tesser J., Modafferi D., Poulakos J., Sun G. (2003). Anakinra, a recombinant human interleukin-1 receptor antagonist (r-metHuIL-1ra), in patients with rheumatoid arthritis: A large, international, multicenter, placebo-controlled trial. Arthritis Rheum..

[47] Kyriazopoulou E., Poulakou G., Milionis H., Metallidis S., Adamis G., Tsiakos K. (2021). Early treatment of COVID-19 with anakinra guided by soluble urokinase plasminogen receptor plasma levels: A double-blind, randomized controlled phase 3 trial. Nat. Med..

[48] Limen R.Y., Sedono R., Sugiarto A., Hariyanto T.I. (2021). Janus kinase (JAK)-inhibitors and coronavirus disease 2019 (Covid-19) outcomes: A systematic review and meta-analysis. Expert Rev. Anti-Infect. Ther..

[49] Jorgensen S.C.J., Tse C.L.Y., Burry L., Dresser L.D. (2020). Baricitinib: A Review of Pharmacology, Safety, and Emerging Clinical Experience in COVID-19. Pharmacotherapy.

[50] Marconi V.C., Ramanan A.V., de Bono S., E Kartman C., Krishnan V., Liao R., Piruzeli M.L.B., Goldman J.D., Alatorre-Alexander J., Pellegrini R.D.C. (2021). Efficacy and safety of baricitinib for the treatment of hospitalised adults with COVID-19 (COV-BARRIER): A randomised, double-blind, parallel-group, placebo-controlled phase 3 trial. Lancet Respir. Med..

[51] Beigel J.H., Tomashek K.M., Dodd L.E., Mehta A.K., Zingman B.S., Kalil A.C., Hohmann E., Chu H.Y., Luetkemeyer A., Kline S. (2020). Remdesivir for the Treatment of COVID-19—Preliminary report. N. Engl. J. Med..

[52] Angamo M.T., Mohammed M.A., Peterson G.M. (2021). Efficacy and safety of remdesivir in hospitalised COVID-19 patients: A systematic review and meta-analysis. Infection.

[53] Takahashi T., Luzum J.A. (2020). Pharmacogenomics of COVID-19 therapies. NPJ Genom. Med..

[54] Carlson M.K., Gleason P.P., Sen S. (2001). Elevation of Hepatic Transaminases after Enoxaparin Use: Case Report and Review of Unfractionated and Low-Molecular-Weight Heparin-Induced Hepatotoxicity. Pharmacother. J. Hum. Pharmacol. Drug Ther..

[55] Yang X., Li N., Guo T., Guan X., Tan J., Gao X., Wu Y., Jia L., Gu M., Hua L. (2020). Comparison of the Effects of Low-Molecular-Weight Heparin and Fondaparinux on Liver Function in Patients With Pulmonary Embolism. J. Clin. Pharmacol..

[56] Hahn K.J., Morales S.J., Lewis J.H. (2015). Enoxaparin-Induced Liver Injury: Case Report and Review of the Literature and FDA Adverse Event Reporting System (FAERS). Drug Saf.-Case Rep..

[57] Harrill A., Roach J., Fier I., Eaddy J.S., Kurtz C.L., Antoine D.J., Spencer D.M., Kishimoto T.K., Pisetsky D.S., Park B.K. (2012). The Effects of Heparins on the Liver: Application of Mechanistic Serum Biomarkers in a Randomized Study in Healthy Volunteers. Clin. Pharmacol. Ther..

[58] Arora N., Goldhaber S.Z. (2006). Anticoagulants and transaminase elevation. Circulation.

[59] Lawler P.R., Goligher E.C., Berger J.S., Neal M.D. (2021). Therapeutic Anticoagulation with Heparin in Noncritically Ill Patients with Covid-19. N. Engl. J. Med..

[60] Goligher E.C., Bradbury C.A. (2021). Therapeutic Anticoagulation with Heparin in Critically Ill Patients with Covid-19. N. Engl. J. Med..

[61] Mazloomzadeh S., Khaleghparast S., Ghadrdoost B., Mousavizadeh M., Baay M.R., Noohi F., Sharifnia H., Ahmadi A., Tavan S., Alamdari N.M. (2021). Effect of Intermediate-Dose vs. Standard-Dose Prophylactic Anticoagulation on Thrombotic Events, Extracorporeal Membrane Oxygenation Treatment, or Mortality Among Patients With COVID-19 Admitted to the Intensive Care Unit: The INSPIRATION Randomized Clinical Trial. JAMA.

[62] Sholzberg M., Tang G.H., Rahhal H., AlHamzah M., Kreuziger L.B., Áinle F.N., Alomran F., Alayed K., Alsheef M., AlSumait F. (2021). Effectiveness of therapeutic heparin versus prophylactic heparin on death, mechanical ventilation, or intensive care unit admission in moderately ill patients with covid-19 admitted to hospital: RAPID randomised clinical trial. BMJ.

[63] Team D.T. (2020). EMA advice on the use of NSAIDs for COVID-19. Drug Ther. Bull..

[64] Micallef J., Soeiro T., Jonville-Béra A.P. (2020). Non-steroidal anti-inflammatory drugs, pharmacology, and COVID-19 infection. Therapie.

[65] Licata A., Minissale M.G., Calvaruso V., Craxì A. (2017). A focus on epidemiology of drug-induced liver injury: Analysis of a prospective cohort. Eur. Rev. Med. Pharmacol. Sci..

[66] Przybyła G.W., Szychowski K.A., Gmiński J. (2020). Paracetamol–An old drug with new mechanisms of action. Clin. Exp. Pharmacol. Physiol..

[67] Fisher E.S., Curry S.C. (2019). Evaluation and treatment of acetaminophen toxicity. Adv. Pharmacol..

[68] Gulmez S.E., Unal U.S., Lassalle R., Chartier A., Grolleau A., Moore N. (2018). Risk of hospital admission for liver injury in users of NSAIDs and nonoverdose paracetamol: Preliminary results from the EPIHAM study. Pharmacoepidemiol. Drug Saf..

[69] Langford B.J., So M., Raybardhan S., Leung V., Soucy J.P., Westwood D., Daneman N., MacFadden D.R. (2021). Antibiotic prescribing in patients with COVID-19: Rapid review and meta-analysis. Clin. Microbiol. Infect..

[70] Chang C.Y., Schiano T.D. (2007). Review article: Drug hepatotoxicity. Aliment. Pharmacol. Ther..

[71] Pani A., Lauriola M., Romandini A., Scaglione F. (2020). Macrolides and viral infections: Focus on azithromycin in COVID-19 pathology. Int. J. Antimicrob. Agents.

[72] Lin S.-J., Kuo M.-L., Hsiao H.-S., Lee P.-T. (2016). Azithromycin modulates immune response of human monocyte-derived dendritic cells and CD4+ T cells. Int. Immunopharmacol..

[73] Gautret P., Lagier J.C., Parola P., Hoang V.T., Meddeb L., Mailhe M., Doudier B., Courjon J., Giordanengo V., Vieira V.E. (2020). Hydroxychloroquine and azithromycin as a treatment of COVID-19: Results of an open-label non-randomized clinical trial. Int. J. Antimicrob. Agents.

[74] Cavalcanti A.B., Zampieri F.G., Rosa R.G., Azevedo L.C., Veiga V.C., Avezum A., Damiani L.P., Marcadenti A., Kawano-Dourado L., Lisboa T. (2020). Hydroxychloroquine with or without Azithromycin in Mild-to-Moderate COVID-19. N. Engl. J. Med..

[75] Molina J., Delaugerre C., Le Goff J., Mela-Lima B., Ponscarme D., Goldwirt L., de Castro N. (2020). No evidence of rapid antiviral clearance or clinical benefit with the combination of hydroxychloroquine and azithromycin in patients with severe COVID-19 infection. Med. Mal. Infect..

[76] Sultana J., Cutroneo P.M., Crisafulli S., Puglisi G., Caramori G., Trifirò G. (2020). Azithromycin in COVID-19 Patients: Pharmacological Mechanism, Clinical Evidence and Prescribing Guidelines. Drug Saf..

[77] AIFA (2020). Azitromicina Nella Terapia dei Pazienti Adulti Con COVID-19. https://www.aifa.gov.it/documents/20142/1123276/azitromicina_08.04.2020.pdf/951fa605-0bf9-3882-ae2f-15128fe97a1b.

[78] Sekhavati E., Jafari F., SeyedAlinaghi S., Jamalimoghadamsiahkali S., Sadr S., Tabarestani M., Pirhayati M., Zendehdel A., Manafi N., Hajiabdolbaghi M. (2020). Safety and effectiveness of azithromycin in patients with COVID-19: An open-label randomised trial. Int. J. Antimicrob. Agents.

[79] Martinez M.A., Vuppalanchi R., Fontana R.J., Stolz A., Kleiner D.E., Hayashi P.H., Gu J., Hoofnagle J.H., Chalasani N. (2015). Clinical and histologic features of azithromycin-induced liver injury. Clin. Gastroenterol. Hepatol..

[80] Azithromycin (2012). LiverTox: Clinical and Research Information on Drug-Induced Liver Injury.

[81] Cammarota G., Esposito T., Azzolina D., Cosentini R., Menzella F., Aliberti S., Coppadoro A., Bellani G., Foti G., Grasselli G. (2021). Noninvasive respiratory support outside the intensive care unit for acute respiratory failure related to coronavirus-19 disease: A systematic review and meta-analysis. Crit. Care.

[82] Grieco D.L., Menga L.S., Cesarano M., Rosà T., Spadaro S., Bitondo M.M., Montomoli J., Falò G., Tonetti T., Cutuli S.L. (2021). Effect of Helmet Noninvasive Ventilation vs. High-Flow Nasal Oxygen on Days Free of Respiratory Support in Patients With COVID-19 and Moderate to Severe Hypoxemic Respiratory Failure: The HENIVOT Randomized Clinical Trial. JAMA.

[83] Gattinoni L., Gattarello S., Steinberg I., Busana M., Palermo P., Lazzari S., Romitti F., Quintel M., Meissner K., Marini J.J. (2021). COVID-19 pneumonia: Pathophysiology and management. Eur. Respir. Rev..

[84] Grasselli G., Zangrillo A., Zanella A., Antonelli M., Cabrini L., Castelli A., Cereda D., Coluccello A., Foti G., Fumagalli R. (2020). Baseline Characteristics and Outcomes of 1591 Patients Infected With SARS-CoV-2 Admitted to ICUs of the Lombardy Region, Italy. JAMA.

[85] Radovanovic D., Pini S., Franceschi E., Pecis M., Airoldi A., Rizzi M., Santus P. (2021). Characteristics and outcomes in hospitalized COVID-19 patients during the first 28 days of the spring and autumn pandemic waves in Milan: An observational prospective study. Respir. Med..

[86] Matuschak G.M. (1994). Liver-lung interactions in critical illness. New horizons.

[87] Kredel M., Muellenbach R.M., Brock R.W., Wilckens H.-H., Brederlau J., Roewer N., Wunder C. (2007). Liver dysfunction after lung recruitment manoeuvres during pressure-controlled ventilation in experimental acute respiratory distress. Crit. Care.

[88] Träger K., Radermacher P., Georgieff M. (1996). PEEP and hepatic metabolic performance in septic shock. Intensive care medicine. Intensive Care Med..

[89] Brienza N., Dalfino L., Cinnella G., Diele C., Bruno F., Fiore T. (2006). Jaundice in critical illness: Promoting factors of a concealed reality. Intensiv. Care Med..

[90] Morris C.A., Avorn J. (2003). Internet marketing of herbal products. JAMA.

[91] Alyami H.S., Orabi M.A., Aldhabbah F.M., Alturki H.N., Aburas W.I., Alfayez A.I., Alharbi A.S., Almasuood R.A., Alsuhaibani N.A. (2020). Knowledge about COVID-19 and beliefs about and use of herbal products during the COVID-19 pandemic: A cross-sectional study in Saudi Arabia. Saudi Pharm. J..

[92] Plášek J., Gumulec J., Máca J., Škarda J., Procházka V., Grézl T., Václavík J. (2021). COVID-19 Associated Coagulopathy: Mechanisms and Host-Directed Treatment. Am. J. Med Sci..

[93] Chong W.H., Saha B.K., Ramani A., Chopra A. (2021). State-of-the-art review of secondary pulmonary infections in patients with COVID-19 pneumonia. Infection.

[94] Torres A., Niederman M.S., Chastre J., Ewig S., Fernandez-Vandellos P., Hanberger H., Kollef M., Bassi G.L., Luna C.M., Martin-Loeches I. (2017). International ERS/ESICM/ESCMID/ALAT guidelines for the management of hospital-acquired pneumonia and ventilator-associated pneumonia: Guidelines for the management of hospital-acquired pneumonia (HAP)/ventilator-associated pneumonia (VAP) of the European Respiratory Society (ERS), European Society of Intensive Care Medicine (ESICM), European Society of Clinical Microbiology and Infectious Diseases (ESCMID) and Asociación Latinoamericana del Tórax (ALAT). Eur. Respir. J..

